# Are Point Mutations in HMG-CoA Reductases (Hmg1 and Hmg2) a Step towards Azole Resistance in *Aspergillus fumigatus*?

**DOI:** 10.3390/molecules26195975

**Published:** 2021-10-01

**Authors:** Irene Gonzalez-Jimenez, Jose Lucio, Alejandra Roldan, Laura Alcazar-Fuoli, Emilia Mellado

**Affiliations:** 1 Mycology Reference Laboratory, National Centre for Microbiology, Instituto de Salud Carlos III (ISCIII), 28220 Majadahonda, Madrid, Spain; irene.gonzalez@isciii.es (I.G.-J.); jose.lucio@isciii.es (J.L.); alex7799roldan@gmail.com (A.R.); lalcazar@isciii.es (L.A.-F.); 2 Spanish Network for Research in Infectious Diseases (REIPI RD16/CIII/0004/0003), ISCIII, 28220 Majadahonda, Madrid, Spain

**Keywords:** *Aspergillus fumigatus*, *hmg1*, *hmg2*, *cyp51A*, azole resistance mechanisms

## Abstract

Invasive aspergillosis, mainly caused by *Aspergillus* *fumigatus*, can lead to severe clinical outcomes in immunocompromised individuals. Antifungal treatment, based on the use of azoles, is crucial to increase survival rates. However, the recent emergence of azole-resistant *A. fumigatus* isolates is affecting the efficacy of the clinical therapy and lowering the success rate of azole strategies against aspergillosis. Azole resistance mechanisms described to date are mainly associated with mutations in the azole target gene *cyp51A* that entail structural changes in Cyp51A or overexpression of the gene. However, strains lacking *cyp51A* modifications but resistant to clinical azoles have recently been detected. Some genes have been proposed as new players in azole resistance. In this study, the gene *hmg1*, recently related to azole resistance, and its paralogue *hmg2* were studied in a collection of fifteen azole-resistant strains without *cyp51A* modifications. Both genes encode HMG-CoA reductases and are involved in the ergosterol biosynthesis. Several mutations located in the sterol sensing domain (SSD) of Hmg1 (D242Y, G307D/S, P309L, K319Q, Y368H, F390L and I412T) and Hmg2 (I235S, V303A, I312S, I360F and V397C) were detected. The role of these mutations in conferring azole resistance is discussed in this work.

## 1. Introduction

*Aspergillus fumigatus* is one of the most ubiquitous saprophytic fungi producing airborne conidia [1]. The primary route of human infection is the inhalation of these conidia that can lead to a wide range of clinical manifestations, named aspergillosis, that cause minor to life-threatening pathologies depending on the immunological status of the patient [2,3]. Mortality rates are variable depending on the severity of the pathology. In invasive aspergillosis (IA), the most severe form of aspergillosis, mortality can reach up to 90% in some patient groups [4]. The treatment options for aspergillosis are limited to three classes of antifungal drugs: azoles, echinocandins and polyenes. Currently, triazole drugs are the first-line treatment and prophylaxis against IA and other *Aspergillus*-related lung diseases [5,6]. *A. fumigatus* azole drug resistance is increasing worldwide and is reducing the therapeutic options for patients infected by azole-resistant *A. fumigatus* (ARAF) strains [7,8]. ARAF strains can emerge by two different routes: a medical route, where resistant isolates are selected inside the host in patients under long-term azole therapy [9]; and an environmental route in which resistance is acquired through the use of demethylation inhibitors (DMIs) to prevent fungal growth in crops, generating cross-resistance with medical triazoles due to the similar structure of both drug classes [10,11].

Clinical triazole drugs, including voriconazole (VCZ), itraconazol (ITC), posaconazole (POS) and isavuconazole (ISV), target the sterol 14-α demethylase, a key enzyme in the ergosterol biosynthesis pathway, encoded by the gene *cyp51A* and its homologue *cyp51B* [12,13]. Until recently, almost all resistance mechanisms found in ARAF strains were based on mutations in the azole target *cyp51A* [3,14,15,16]; nevertheless, ARAF strains without *cyp51A* modifications have started to be studied recently [17,18]. These isolates seem to be resistant to all clinical azole drugs without any *cyp51A* modification [19]. The isolation of these strains is relatively common in some geographic regions such as Japan or the United States, where 40% and 60% of all azole-resistant isolates, respectively, do not show any *cyp51A*-related resistance mechanism [17,20]; however, studies in the Netherlands place this prevalence at 15% [21].

Several genes with different implications in the fungal cell have been proposed as *A. fumigatus* azole-resistance candidates. Transcriptional factors such as *hapE* or ABC transporters including *crd1B*, *mdr1* or *mdr2* are some examples [19]. Among the possible candidates, the *hmg1* gene has been increasingly studied and proposed as responsible for azole resistance in wild-type *cyp51A* ARAF strains [17]. *Hmg1* encodes a HMG-CoA reductase (HMGR), an enzyme that catalyzes the reduction of HMG-CoA to mevalonic acid, a rate-limiting step in the ergosterol biosynthesis pathway [22]. Several mutations in Hmg1, that seem to be related to multiazole resistance, have already been described (Table 1) [17,18,23,24,25,26,27,28,29,30,31,32]. Among them, the mutation S269F (*hmg*1^S269F^) has been identified and related to an increased level of ergosterol in cell walls, suggesting its link with azole resistance [17]. Most of the amino acid changes described in Hmg1 (F262del, S269F, S305P, G307R/D, E307D, P309L, F390Y, L413P) are located within the sterol sensing domain (SSD), a conserved transmembrane motif anchored to the endoplasmic reticulum involved in regulation of the sterol synthesis pathway but not in the catalytic activity of the enzyme [33] (Table 1). Other amino acid changes located outside the SSD, such as E105K, G466V, S541G, or H564Y have also been described, but since they were found in azole-susceptible strains as well as in azole-resistant ones, they do not seem to be implicated in azole resistance [17,22,26,29].

Usually, mutations in Hmg1 have been related to azole resistance as the only azole resistance mechanism. However, it is also true that quite frequently these mutations are present in strains with Cyp51A mutations [17,22,24] or, in Cyp51B, as recently described [34]. In *A. fumigatus*, besides Hmg1, a second HMG-CoA reductase enzyme (Hmg2) is present as happens with other enzymes of the ergosterol biosynthesis pathway [35], although its relation with antifungal resistance has not been studied yet.

In this study, we analyze 15 pan-azole-resistant *A. fumigatus* strains without mutations in the azole target gene (*cyp51A*). We focus on looking for single nucleotide polymorphisms (SNPs) in *hmg1* and *hmg2* that could explain the azole resistance phenotype of these isolates. Strains with mutations in Hmg1 or Hmg2 were further analyzed in order to determine the possible implication of these alterations in the development of azole resistance.

## 2. Results

### 2.1. AFST and Strain Genotyping

MICs to clinical antifungals were performed in a set of 15 *A. fumigatus* clinical strains. Susceptibility results were considered following the EUCAST methodology as referred in the material and methods section. Strains were considered resistant with MICs over 1 mg/L to ITC and VCZ, 2 mg/L to ISV and 0.25 mg/L for POS according to the EUCAST breakpoints (https://www.eucast.org/astoffungi/clinicalbreakpointsforantifungals/, accessed on 2 September 2021). All isolates included in this study were susceptible to AmB and resistant to the four clinical azoles tested, with MICs above the clinical breakpoints (Table 2). Genotyping results following the TRESPERG method [36] for all the isolates included in our study are detailed in Table 2. All isolates have different genotypes, which indicates that they are not genetically related and thus the underlying azole resistance mechanism in all the strains will likely have evolved individually in each of them.

### 2.2. Sequence Analysis of Cyp51A, Cyp51B, Hmg1 and Hmg2 Genes in Azole-Resistant Strains

The analysis of *cyp51A* and its promoter revealed that none of the isolates harbored any mutation in the promoter or the coding sequence of the gene, indicating that the resistance to clinical azoles observed in these strains is not a consequence of a *cyp51A*-related resistance mechanism (Table 2). The analysis of *cyp51B* showed that only one strain (CM9640) had a mutation G457S in Cyp51B, and the remaining strains had wild-type *cyp51B* sequences. Information about the implication of the amino acid change G457S in Cyp51B has been previously published, also including information about the F390L mutation in Hmg1 that was present in the same strain [34].

Apart from strain CM9640, three strains had mutations in Hmg1: G307D in strain CM3720, P309L in CM7510 and D242Y in strain CM9471. All of them were found within the SSD of the Hmg1 protein structure. In Hmg2 some amino acid substitutions were also detected in the SSD, substitution I235S was harbored by three strains, CM1910, CM8900 and CM9709, the last also harboring a Q533H mutation. Strain CM3720 harbored an I312S amino acid change and strain CM9956 had a stop codon at position E415 (Table 2).

### 2.3. Hmg1, Hmg2, Cyp51A and Cyp51B Expression Analysis with and without Azole Induction

**Figure 1:**
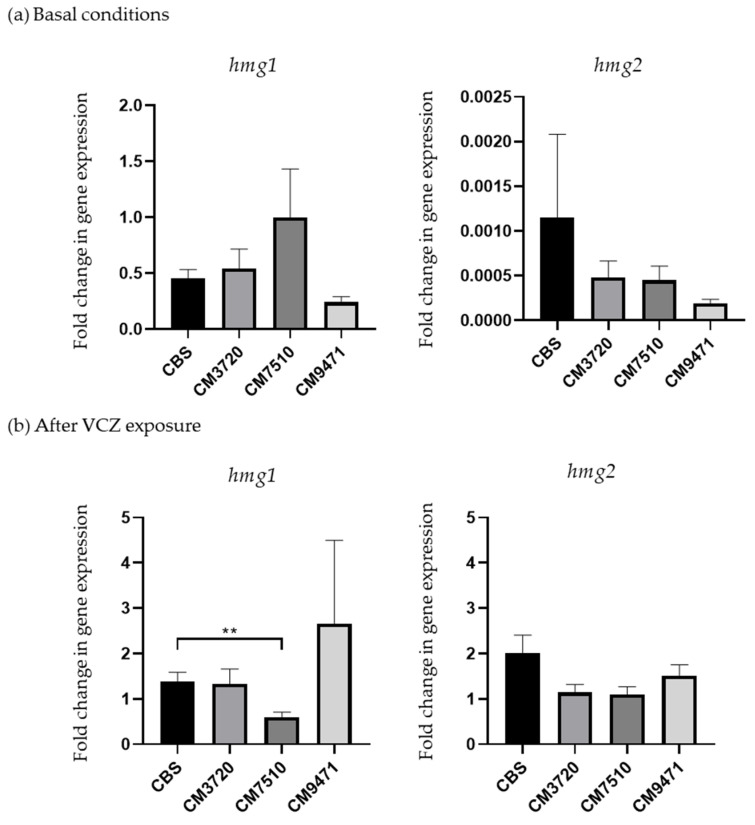
Expression analysis of *hmg1* and *hmg2* genes in a wild-type *A. fumigatus* strain (CBS) and three *hmg1/2*-mutated strains: CM3720 (G307D, I312S), CM7510 (P309L) and CM9471 (D242Y). (**a**) Expression after overnight growth of the strains in basal conditions. (**b**) Relative expression of *hmg1* and *hmg2* genes in strains after one hour of VCZ exposure relative to basal conditions. ** *p* < 0.01.

Expression analysis for genes *hmg1*, *hmg2*, *cyp51A* and *cyp51B* were carried out through RT-qPCR in *A. fumigatus* strains CBS (wild-type control strain) and the strains harboring mutations in Hmg1/Hmg2: CM3720 (*hmg1*^G307D^ and *hmg2*^I312S^), CM7510 (*hmg1*^P309L^) and CM9471 (*hmg1*^D242Y^) (Figure 1). Gene expression was evaluated when strains were grown in the absence and presence of VCZ. Results from the expression analysis show that *hmg1* and *hmg2* genes were expressed in basal conditions and when VCZ was added (Figure 1). We confirmed that the expression levels for genes *cyp51A* and *cyp51B* did not show any significant difference between the wild-type and the *hmg1*-mutated strains in any of the conditions tested (data not shown).

*Hmg1* shows similar expression levels in the different strains and the different conditions tested. An increase of expression of *hmg1* was observed in strain CM7510 as well as a decrease in CM9471 in basal expression conditions, but none of the changes were statistically significant. Only the expression of *hmg1* in strain CM7510 had a statistically significant difference (*p* < 0.01) compared with the wild type strain CBS, showing a reduction in the VCZ-treated sample (Figure 1b).

The gene *hmg2* in the wild-type strain shows very low levels of expression in basal conditions or stimulated with the presence of VCZ. No increase of expression was observed in the strains mutated in Hmg1 in any condition. In addition, strain CM3720, which also harbors an I312S mutation in Hmg2, did not show any increased *hmg2* production in comparison with the wild-type.

### 2.4. Identifying Hmg1 and Hmg2 Mutations in an A. fumigatus Strain Collection by Whole Genome Sequencing (WGS)

To expand our study, a database of 170 whole genomes of *A. fumigatus* strains was used to analyze polymorphisms in the sequence of both *hmg1* and *hmg2* genes. The *A. fumigatus* genomes database includes azole-susceptible and azole-resistant strains from different geographical origins with very diverse azole resistance mechanisms [34,37]. In *hmg1*, four amino acid substitutions were found located within the SSD of the protein (Table 3): G307S in four azole-resistant strains with underlying Cyp51A mutations, K319Q and Y368H in azole-resistant strains with Cyp51A azole resistance mechanisms and I412T in two azole-susceptible strains. Results from the analysis of the complete sequence of *hmg1* have been previously published [34] and are summarized in Table S1.

As happens with *hmg1*, *hmg2* is a highly polymorphic gene in its whole sequence, not only in the SSD. A total of 19 amino acid changes were found in azole-susceptible and azole-resistant strains (Table S2). Five mutations were found in the region of the SSD (Table 4): I235S in three strains including two azole-susceptible and one azole-resistant isolates; V303A in five azole-susceptible strains and ten azole-resistant strains with underlying Cyp51A resistance mechanisms; I312S in four strains including three azole-susceptible and one azole-resistant; I360F in ten azole-resistant strains with Cyp51A mutations and Y397C in one azole-susceptible strain.

### 2.5. Sequence Analysis of Hmg1 and Hmg2 Genes and Protein Structural Analysis

**Figure 2:**
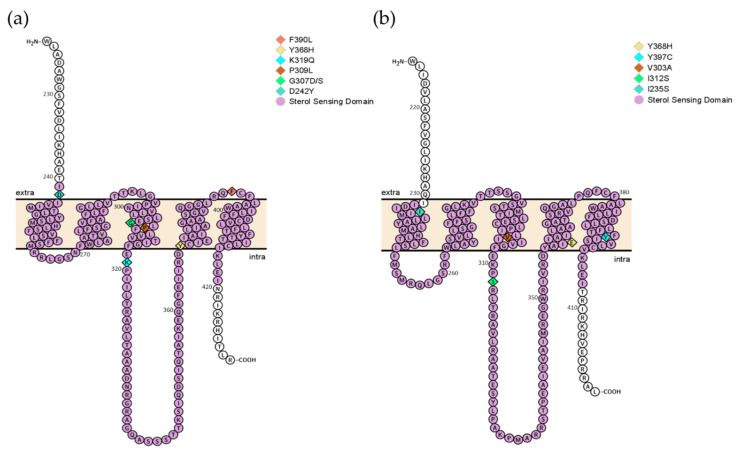
Secondary structure of the sterol sensing domain (SSD) regions of the proteins Hmg1 (**a**) and Hmg2 (**b**). Purple residues are indicating the SSD with the five transmembrane regions. The section of the Hmg1 and Hmg2 proteins represented here are truncated 18 amino acids before the SSD and 10 and 13, respectively, after. The amino acids marked in different colors and rhomboid shape represent the ones in which mutations have been described in this work.

The gene *hmg1* is 3570 bp long, encoding an HMGR of 1130 amino acids (aa) (Hmg1). Hmg1 proteins from both reference strains (AF293 and A1163) are 99.8% identical with only two amino acid differences (S212P and Y564H). The *hmg2* gene is 3378 bp long and also encodes an HMGR of 1062 aa (Hmg2). The Hmg2 proteins from both reference strains are 99.91% identical with only one different amino acid (A100V). Hmg1 and Hmg2 share 56% of identity along the full protein and 61% in the SSD. HMGRs are bound to the endoplasmic reticulum membranes through their N-terminal domains that contain eight transmembrane (TM) helices. On the other end, the C-terminal domain of the protein projects into the cytosol and contains the entire reductase catalytic activity [38,39,40]. Both proteins share a sterol-sensing domain (SSD), normally displayed in membrane proteins, responsible for sterol regulation. The SSD normally consists of five consecutive membrane-spanning domains (Figure S1). In Hmg1, the SSD domain comprises a 178 aa region between the aa positions 241–419 and in Hmg2 this SSD region, consisting of 175 aa, is located between the positions 232–407 of the protein (Figure 2).

### 2.6. Mutations outside the SSD of Hmg1 Do Not Contribute to Azole Resistance in A. fumigatus

While trying to demonstrate the importance of different Hmg1 mutations, we analyzed three clinical *A. fumigatus* isolates, CM9114 (2017), CM9501 (2018) and CM9551 (2019), obtained from three independent patients in the same geographic region (Barcelona, Spain) but isolated in consecutive years. All of them were resistant to clinical azoles with the exact same MIC profile and the same genotype (Table 5). We amplified and sequenced the genes *cyp51A, hmg1* and *hmg2* searching for possible azole resistance mechanisms, finding that all three isolates had the same G54R substitution in Cyp51A but different mutations in Hmg1. Strain CM9501 did not have any mutation in *hmg1*, strain CM9114 had an Y564H substitution which has been previously described [17,41], and strain CM9551 had a g779a polymorphism. The amino acid position Y564 is located outside the SSD of Hmg1 and the nucleotide g799 is located within an intron that is located inside the region of the SSD of Hmg1. The MIC profile of the three strains did not change due to the *hmg1* mutations present in two of them.

## 3. Discussion

Resistance to azoles in *A. fumigatus* is an increasing worldwide issue that reduces therapeutic options for patients suffering from aspergillosis [42]. To date, the most common mechanisms of resistance are mutations in the azole target, Cyp51A. However, the isolation of multi azole-resistant *A. fumigatus* strains without *cyp51A* modifications has increased, implying that other resistance mechanisms are emerging [43]. A similar trend has been detected in our laboratory, opening a new field for investigating novel azole resistance mechanisms.

Mutations located specifically in the sterol-sensing domain (SSD) of the Hmg1 protein have been recently proposed as a possible azole resistance mechanism in strains with wild-type *cyp51A*. As the SSD domain is involved in binding to the endoplasmic reticulum membrane and ergosterol homeostasis, the change of specific amino acids could disrupt the function of the enzyme [44]. All of the strains included in this study were resistant to the main clinical triazole drugs, with different genotypes, and none of them harbored a Cyp51A resistance mechanism. Only one strain had a mutation G457S in Cyp51B that has been independently studied [34]. The amino acid substitutions D242Y in strain CM9471, G307D in CM3720, P309L in CM7510 and F390L in CM9640 were detected in Hmg1. The role of G457S in Cyp51B and F390L in Hmg1 in inducing triazole resistance, as mentioned before, has been previously published [34]. In addition, a recent study introduced the mutation G457S in a wild-type strain, detecting higher levels of resistance to VCZ but not to ITC or POS, concluding that the mutation in Cyp51B conferred resistance to VCZ and that the resistance to ITC and POS should be derived from the F390L in Hmg1 [45]. In Hmg2, the substitution I235S was harbored by three *A. fumigatus* strains (CM1910, CM8900 and CM9709) and the mutation I312S was present in one strain (CM3720). Other mutations were found but located outside the SSD of Hmg2.

Both genes *hmg1* and *hmg2* are expressed in basal conditions although the expression levels of *hmg1* are much higher than *hmg2*, as has been previously reported in other fungal species such as *Saccharomyces cerevisiae* and also in *A. fumigatus* [46,47]. The remarkably lower levels of expression shown by the *hmg2* gene both in wild-type and mutated strains under the different conditions tested leads us to think that *hmg2* does not have a relevant role in the azole resistance phenotype of the strain. However, other studies [48] have described an increased up-regulation of *hmg2* under ITC exposure. Thus, the role of *hmg2* in azole resistance remains controversial. Apart from that, *hmg1*-mutated strains did not show a clear pattern of different regulation in the strains harboring amino acid substitutions in Hmg1 when they were under VCZ exposure or untreated. For this reason, the implication of the mutations in Hmg1 do not seem to be linked to changes in expression of the *hmg1* gene itself or other genes involved in the ergosterol biosynthesis pathway, as is the case of *cyp51A* and *cyp51B* whose expression was not affected in the mutated strains.

A collection of 170 *A. fumigatus* genomes was analyzed by WGS in order to study the diversity of Hmg1 and Hmg2 in a larger population, finding several polymorphisms and non-synonymous mutations. However, the majority of these mutations were also found in *A. fumigatus* azole-susceptible strains, which eliminates their direct role in the azole resistance phenotype. In addition, many strains also had a Cyp51A resistance mechanism, which was the main cause of their azole resistance pattern. Previous studies have found several amino acid substitutions within the SSD of Hmg1 but mostly present in azole-susceptible strains or strains with other resistance mechanisms (Table S1) [29,34]. Only the above-mentioned mutations D242Y, G307D and P309L were detected in azole-resistant strains without any other known resistance mechanism and, for this reason, their possible implication in conferring the resistant phenotype is proposed. Regarding Hmg2, the substitution I235S was also present in one azole-resistant strain with an M220V substitution in Cyp51A and azole-susceptible strains, and therefore it does not seem to be the resistance mechanism prevailing in those wild-type Cyp51A strains. Mutations V303A, I312S and Y397C were only harbored by azole-susceptible strains so their implication in azole resistance is also disregarded. In addition, the mutation I360F was detected in azole-resistant isolates but with well characterized Cyp51A resistance mechanisms (TR_34_/L98H, G138C or M220T). This coexistence of mutations in Hmg1/2 and Cyp51A is an interesting finding that will be discussed later.

The fact that three isogenic strains with different Hmg1 substitutions, one of them with a substitution located outside the SSD, had the same MICs to clinical azoles, suggests that mutations outside the SSD of Hmg1 do not affect the azole susceptibility of *A. fumigatus*, nor does the polymorphism located within the second intron of *hmg1* that has been detected in several strains including azole-susceptible and resistant *A. fumigatus* strains (data not shown). In addition, Hawigara et al. [17] detected the same H564Y in one azole-susceptible isolate.

To date, the implication of Hmg1 mutations in *A. fumigatus* azole resistance remains controversial. Although many azole-resistant strains have been found carrying Hmg1 mutations (Table 1), their role in azole resistance, by reintroducing the mutated copy of Hmg1 into an Hmg1 wild-type *A. fumigatus* strain, has only been demonstrated in a few of them (Table 6) and sometimes with contradictory results [17,23,25,30].

Initially, Losada et al. [31] identified Hmg1 as another player in azole resistance in an experiment involving sequential in vitro exposure of *A. fumigatus* to various azole compounds. Using this methodology, several azole-resistant isolates emerged from which 71% harbored at least one mutation in *cyp51A* and 38% in the *hmg1* gene. A combination of mutations in both genes was detected in four isolates. Following a similar strategy, Zhang et al. [33] evidenced that *hmg1* mutations appear as a general adaptation mechanism to triazole pressure before *cyp51A* modifications, associated with increased triazole resistance. This work, together with these previous studies, show that some of the strains with triazole resistance phenotypes and *hmg1* mutations also had modifications in *cyp51A* in combination [17,23,26,28,41]. As shown in our results, mutation in the paralogous gene *hmg2* could also be contributing to the adaptive mechanism similar to *hmg1*, although this hypothesis is less clear.

Previous studies [18,23,28,29] have acknowledged the relation between mutations in Hmg1 and azole resistance in strains without Cyp51A modifications. By using the CRISPR-Cas9 method, Rybak et al. [23] were able to restore the susceptibility to triazoles in strains harboring Hmg1 mutations F262del, S305P and I412S. The amino acid changes found in our study are located in positions near the ones they studied (D242, G307 and P309), which leads us to think that these mutations can also be the main cause of azole resistance in the strains from our study. In addition, Losada et al. [31] have analyzed *A. fumigatus* strains selected with voriconazole and found *hmg1* mutants with VCZ resistance. Furthermore, they described that strains with mutations in both Cyp51A and the SSD of HMGR enzymes conferred full resistance to voriconazole, but each mutation separately was sufficient for high-level azole resistance. Along the same line of research, Mortensen et al. [9] isolated isogenic strains from the same patient in successive years. The strain isolated in the first place was susceptible to azoles and did not have any *cyp51A* alteration. A later isogenic isolate was resistant to azoles and with no mutation in *cyp51A*. Lastly, a strain resistant to azoles with *cyp51A* alterations and the same genotype as the other two was isolated. This suggests a possible alteration before the ones in *cyp51A* and which can predispose the *cyp51A* alterations to appear. Until recently, the study of resistance in *A. fumigatus* was focused on studying *cyp51A,* and *hmg1* was rarely considered, so strains that had *cyp51A* mutations might have had alterations in *hmg1* before the development of the mutations in *cyp51A*. In a previous study [34], this theory was also proposed, showing a large collection of *A. fumigatus* strains that harbored mutations in both genes, *cyp51A* and *hmg1*. Other studies relative to the relation of *hmg1* with azole resistance [23] show that strains harboring mutations in *hmg1* do not have MICs to azoles as high as the strains harboring mutations in *cyp51A*, reinforcing the theory that a mutation in *hmg1* could decrease the susceptibility against these drugs, but not enough to confer complete resistance by itself. Only in a few cases a direct effect of the Hmg1 mutation in azole resistance has been demonstrated, so it is possible that only changes in specific residues could be directly responsible for a full azole resistance phenotype. To date, only mutations located in the third membrane-spanning domain seem to be responsible for the azole resistance phenotype. However, in other cases it seems that the mutations at the SSD can involve a step previous to the development of full azole resistance [45]. This implies that studies of each individual residue modification need to be done in order to define their role in azole resistance.

In summary, here we reinforce the theory of a novel mechanism of triazole resistance in *A. fumigatus* based on Hmg1 mutations, which were found in three of 15 ARAF clinical isolates without a known resistance mechanism. The results of our study identify novel mutations in the *A. fumigatus* HMG-CoA reductase gene *hmg1* as determinants of triazole resistance. Nevertheless, the exact mechanism by which mutations in the SSD of the *hmg1* gene cause resistance remains unknown. Hypotheses are focused on the role of the sterol-sensing domain in the negative regulation of *hmg1*, as has been seen in other organisms [43]. The detection of *hmg2* and its modifications in this study is new and opens the possibility of a new resistance mechanism similar to Hmg1 alterations. This is a novel finding that needs more research, as some mutations in Hmg2, such as I360F and V303A could be good candidates to be explored as a contributing resistance mechanisms before Cyp51A mutations and full azole resistance development. No resistance mechanisms were found in the remaining strains under study, which means that other factors still unknown may be contributing to azole resistance, and need to be explored.

## 4. Materials and Methods

### 4.1. Aspergillus fumigatus Strains and Genotyping

A total of 15 azole-resistant clinical strains of *A. fumigatus,* obtained from individual patients over a period of 19 years (2001–2020), were analyzed in this study (Table 2). All of them belong to the strain collection of the Spanish National Center of Microbiology and were mainly isolated in Spain, except the strain CM3720, which was isolated in The Netherlands. All the strains included in this study were genotyped following the previously described typing method TRESPERG (Table 2) [36,49]. The *A. fumigatus* CBS144.89 (A1163) azole-susceptible strain was used as a reference strain for all the experimental procedures.

### 4.2. Antifungal Susceptibility Testing

Antifungal susceptibility testing (AFST) was performed following the broth microdilution method described by the European Committee on Antimicrobial Susceptibility Testing (EUCAST) broth microdilution reference method v3.0 [50]. Antifungals used were amphotericin B (AmB) (Sigma-Aldrich Química, Madrid, Spain), itraconazole (ITC) (Janssen Pharmaceutica, Madrid, Spain), voriconazole (VCZ) (Pfizer SA, Madrid, Spain), posaconazole (POS) (Merck and Co., Inc., Kenilworth, NJ, USA) and isavuconazole (ISV) (BasileaPharmaceutica, Basel, Switzerland (tested from January 2017)). The final concentrations tested ranged from 0.03 to 16 mg/L for AmB and 0.015 to 8 mg/L for the four azoles tested. *A. flavus* ATCC 204304 and *A. fumigatus* ATCC 204305 were used as quality control strains in all tests performed. Minimal inhibitory concentrations (MICs) were visually read after 24 and 48 h of incubation at 37 °C in a humid atmosphere. MICs were performed at least twice for each isolate. Clinical breakpoints for interpreting AFST results established by EUCAST v10.0 [51] were used for classifying the *A. fumigatus* strains as susceptible or resistant. 

### 4.3. Hmg1, Hmg2, Cyp51A and Cyp51B Amplification, PCR Conditions and Sequencing

For the DNA extraction, conidia from each strain were cultured in glucose–yeast extract-peptone (GYEP) liquid medium (0.3% yeast extract, 1% peptone; Difco, Soria Melguizo) with 2% glucose (Sigma-Aldrich, Madrid, Spain) for 24 h at 37 °C. After mechanical disruption of the mycelium by vortex-mixing with glass beads, genomic DNA of isolates was extracted using the phenol-chloroform method [52]. Molecular identification was performed by PCR amplifying and sequencing ITS1-5.8S-ITS2 regions and a portion of the β-tubulin gene [53].

The whole coding sequences from the different target genes under study including the promoter of *cyp51A* were amplified and sequenced. To exclude the possibility that any change identified in the sequences was due to PCR-induced errors, each isolate was independently analyzed twice. Primers used to amplify the sequence of the genes analyzed in this study are listed in Table S1. The PCR reaction mixtures contained 0.5 µM of each primer, 0.2 µM deoxynucleoside triphosphate (Roche, Spain), 5 µL of PCR 10X buffer, 2 mM MgCl_2_, DMSO 5.2%, 2.5 U of TaqDNA polymerase (Applied Biosystems, CA, USA), and 100–200 ng of DNA in a final volume of 50 µL. A DNA 1-kb molecular ladder (Promega, Spain) was used for all electrophoresis analyses. The samples were amplified in a GeneAmp PCR System 9700 (Applied Biosystems) by using the following program parameters: 1 cycle of 5 min at 94 °C and then 35 cycles of 30 s at 94 °C, 45 s at melting temperature specific for each primer pair (Table S3), and 2 min at 72 °C, followed by a final cycle of 5 min at 72 °C. For *hmg1* and *hmg2* primers, P1 and P2 were used to amplify the sterol sensing domain (SSD) and primers from P3 to P10 were used to amplify the whole gene. The amplified products were purified using IllustraExoprostar 1–step (GE Helthcare Life Sciende, UK) and both strands were sequenced with the Big-Dye terminator cycle sequencing kit (Applied Biosystems) following the manufacturer’s instructions, using the primers listed in Table S1. All gene sequences were edited and assembled using the Lasergene software package (DNAStar, Inc., Madison, WI, USA).

### 4.4. RNA Isolation and Reverse Transcription-Quantitative PCR (RT-qPCR)

A concentration of 10^6^ conidia per ml was cultured overnight in 100 mL of minimal medium broth at 35 °C and 150 rpm. Voriconazole at 0.0125 mg/L was added after overnight growth during 1 h of exposure [54]. Mycelium was filtered using DEPC water (0.1%), a funnel and miracloth paper (CalbiochemR, Merck Millipore, Spain), blot dried, frozen with liquid nitrogen, and ground to powder. RNA was extracted using an RNesay Plant Mini Kit (Qiagen, Spain) following the manufacturer’s instructions and DNA was eliminated using the DNA-*free*^TM^Kit (Invitrogen). RNA concentrations and quality rates were measured using NanoDropOne (Thermo Scientific) and samples were conserved at −80 °C. From the RNA extracted, cDNA was obtained through reverse transcription by using the commercial ImProm-II^TM^ Reverse Transcription System kit (Promega, Spain). The reaction mixtures were prepared following the manufacturer’s instructions: 1 μg of RNA, 1 μg/mL of Oligo dT, 4.5 μg of RNase-free water, 4 μL of ImProm-II Reaction Buffer (5X), 4 μL of MgCl_2_, 1 μL of dNTPs, 0.5 μL of ribonucleases inhibitor (rRNasin^®^) and 1 μL of reverse transcriptase (ImPro-II^TM^) in a final volume of 20 μL. The reverse transcription reaction parameters followed were 5 min at 25 °C, 60 min at 42 °C and 15 min at 70 °C. The final cDNA obtained was diluted (1:5) in RNase-free water.

Genetic expression experiments were performed by quantitative PCR in the CFX90 system (Bio-Rad, Madrid, Spain), using the intercalating agent SYBR Green (Applied Biosystems, CA, USA). Reaction mixture was prepared using FastStart DNA Master SYBR Green containing 0.6 μL of MgCl_2_, 1.6 μL (10 mM) of each primer, 10 μL of 2xSensiMix SYBR Hi-ROX (Roche Diagnostic, Barcelona) and 2 μL of cDNA in a final volume of 20 μL. Before performing the expression analysis experiments, primer conditions were standardized for the quantitative PCR. The PCR conditions were 10 min at 95 °C and 40 cycles of 10 s at 95 °C, 5 s at 58 °C, and 30 s at 72 °C. The *hmg1*, *hmg2*, *cyp51A* and *cyp51B* expression levels were quantified for each strain using the *A. fumigatus* β-tubulin gene (*tub1*, GenBank accession number AY048754) as a reference for gene expression. Primers used to amplify the cDNA from *tub1, hmg1*, *hmg2*, *cyp51A* and *cyp51B* genes are listed in Table S4. Bio-Rad qPCR mixtures were set up with SensiMix SYBR-Hi carboxy-X-rhodamine (Bioline, Spain). Each assay was repeated in triplicate with RNA from three different extractions. Each experiment included standard curves for the target genes (*hmg1*, *hmg2*, *cyp51A* and *cyp51B*) and the reference gene (*tub1*). The efficiencies of PCR amplification of β-tubulin, *hmg1*, *hmg2*, *cyp51A* and *cyp51B* cDNA were calculated from the slopes of the curves given by Bio-Rad CFX manager (version 2.0) software (Bio-Rad Laboratories, Inc, Hercules, CA, USA), and the efficiency values were used to validate each experiment. Fold changes in expression were calculated using the 2^−ΔΔCT^ threshold cycle (CT) method [55]. Statistical analyses were performed with GraphPad Prism, version 5 Project (GraphPad Software, San Diego, CA, USA). The statistical significance of variances between fungal isolates was calculated by using a nonparametric Mann–Whitney *t*-test. A *p* value < 0.01 was considered significant.

### 4.5. Genome Sequence Analysis: Determining Modifications in Genes of Interest

A previous whole genome sequencing (WGS) analysis was performed in our laboratory including a total of 170 strains between the ones from our collection and genomes downloaded from databases [37]. Based on this WGS the genes *cyp51A*,* cyp51B*,* hmg1* and *hmg2* were analyzed in order to find single nucleotide polymorphisms (SNPs). Two reference *A. fumigatus* strains Af293 and Af1163 were used as controls in order to discard SNPs specific to the genome background of the strains; Af293 *hmg*1 (Afu2g03700), *hmg*2 (Afu1g11230), *cyp51A* (Afu4g06890), *cyp51B* (Afu7g03740), and Af1163 *hmg*1 (AFUB_020770), *hmg*2 (AFUB_010660), *cyp51A* (AFUB_063960), *cyp51B* (AFUB_089270).

A 2D protein structural analysis was performed for the Hmg1 and Hmg2 proteins and their SSD using Protter (ETH Zürich). The full coding sequences of *hmg1* and *hmg2* from the reference *A. fumigatus* whole genome sequenced strains, Af293 and A1163, were downloaded from databases (*hmg1*: Afu2g03700 (Af293) and AFUB_010660 (A1163); *hmg2*: Afu1g11230 (Af293) and AFUB_020770 (A1163)) and translated into proteins using the Lasergene software package (DNAStar, Inc., Madison, WI, USA).

## Figures and Tables

**Table 1:** Mutations in the SSD domain of Hmg1 that have been reported to be involved in *A. fumigatus* azole resistance and MICs to azoles and AmB of the strains.

Hmg1 Mutation	MICs (mg/L)					References
	ITC	VCZ	POS	ISV	AmB	
Y250H	>32	4	0.125	-	-	[23]
F261del	4	8	2		2	[17]
F261del	4	8	-	-	-	[24]
F262del	32	8	2	8		[23]
S269Y	8	>8	-	-	-	[24]
S269Y	8	>8	8		1	[17]
S269F	>8	>8	8	-	1	[17]
S269F	>8	>8	4	-	-	[25]
S269P	>1	>1	>1	>1	-	[26]
S269F, F390Y	16	8	2	8	-	[18]
W272C	>16	8	4	8	0.5	[27]
L273F	4	4	-	-	-	[28]
L273F	4	4	-	-	-	[24]
W273S	1	4	0.5	-	-	[29]
L304P	>8	8	1	-	-	[29]
S305P	>8	>8	-	-	-	[28]
S305P	>32	16	1	-	-	[23]
S305P/M220I	>8	>8	8	-	-	[25]
E306K	>16	4	2		0.125	[30]
G307D	>16	8	8	-	-	[29]
G307R	>16	13	2	-	-	[29]
G307D/M220I	>8	>8	>8	-	-	[25]
E307D	-	-	-	-	-	[31]
P320L	8–32	1–4	0.5	-	-	[32]
F390Y	4	8	4	-	1	[17]
F390Y	4	8	-	-	-	[24]
I412S	>32	8–16	0.5–1	-	-	[23]

MIC, minimal inhibitory concentration; ITC, itraconazole; VCZ, voriconazole; POS, posaconazole; ISV, isavuconazole; AmB, amphotericin B.

**Table 2:** Clinical *A. fumigatus* isolates resistant to clinical azoles including information about year of isolation, minimal inhibitory concentrations (MICs) to clinical antifungals, genotype and amino acid substitutions in genes related to azole resistance.

Strain	Isolation Year	Clinical Antifungals MICs (mg/L)					TRESPERG Genotype	Amino Acid Substitutions			
		AmB	ITC	VCZ	POS	ISV		Cyp51A	Cyp51B	Hmg1	Hmg2
ATCC	Ref	0.25–1	0.12–1	0.25–1	0.03–0.25	0.25–1	t01m5.5c03.e11	WT	WT	WT	WT
CBS	Ref	0.25–1	0.12–1	0.25–1	0.03–0.25	0.25–1	t01m5.6c08A.e07	WT	WT	WT	WT
CM1910	2001	0.25–1	>8	2–4	1	ND	t04Am5.3c08A.e07	WT	WT	WT	I235S
CM3720	2005	0.12–0.25	>8	4–8	0.5–1	4–8	t13m7.1c11.e10	WT	WT	G307D	I312S
CM7510	2014	0.25	>8	2–4	0.5	>8	t01m1.1c18.e07	WT	WT	P309L	WT
CM7552	2014	1	>8	1	0.5	ND	t01m5.3c05A.e12	WT	WT	WT	WT
CM7555	2014	0.5	>8	0.25–1	0.5	1	t01m5.3c05A.e11	WT	WT	WT	WT
CM8822	2017	2	4	8	0.5	4	t02m1.1c17.e04	WT	WT	WT	WT
CM8900	2017	0.25–1	1–8	4–>8	0.5	2	t01m1.1c04.e13	WT	WT	WT	I235S
CM9471	2018	0.25	>8	8	0.5	8	t04Am3.4c05A.e07	WT	WT	D242Y	WT
CM9494	2018	0.25	8	2	0.5	4	t01m1.1c18.e07	WT	WT	WT	WT
CM9512	2019	0.25	>8	2	0.5	8	t04Am1.1c08A.e09	WT	WT	WT	WT
CM9640	2019	1	>8	>8	>8	>8	t04Am1.3c08A.e07	WT	G457S	F390L	WT
CM9709	2019	1	2	4	1	4	t03m1.1c08A.e07	WT	WT	WT	I235S, Q533H
CM9735	2019	0.5	8	1	0.5	2	t03m1.1c09.e09	WT	WT	WT	WT
CM9956	2020	1	2	8	0.25	4	t11m1.2c08A.e13	WT	WT	WT	E415Stop
CM9974	2020	0.5	>8	2	0.25	2	t01m5.5c05A.e15	WT	WT	WT	WT

Ref, Reference strains: ATCC 204305, CBS144.89; AmB, Amphotericin B; ITC, itraconazole; VCZ, voriconazole; POS, posaconazole; ISV, isavuconazole. ND: no data.

**Table 3:** Analysis of the Hmg1 amino acid substitutions found in our set of 170 *A. fumigatus* clinical strains. Only substitutions found inside the SSD are listed. Concomitant Cyp51A azole resistance mechanisms are also shown.

Nucleotide (cDNA)	Codon Change	Aa Change	N° of Strains	Azole S	Azole R	Cyp51A Modifications	Percentage (%)
919	Ggc/Agc	G307S	4	0	4	G138C	2.4
955	Aag/Cag	K319Q	1	0	1	M220I/V101F	0.5
1102	Tac/Cac	Y368H	1	0	1	P216L	0.5
1235	aTc/aCc	I412T	2	2	0	--	1.17

aa, amino acid; S, susceptible; R, resistant/resistance.

**Table 4:** Analysis of the SSD Hmg2 amino acid substitutions found in our set of 170 *A. fumigatus* clinical strains. Concomitant Cyp51A azole resistance mechanisms are also shown.

Nucleotide (cDNA)	Codon Change	Aa Change	N° of Strains	Azole S	Azole R	Cyp51A Modifications	Percentage (%)
704	aTc/aGc	I235S	3	2	1	M220V	1.8
908	gTa/gCa	V303A	15	5	10	TR_34_/L98H/+ *	8.8
935	aTc/aGc	I312S	4	3	1	3SNPs	2.4
1078	Att/Ttt	I360F	10	0	10	TR_34_/L98H/+ *	5.9
1190	tAc/tGc	Y397C	1	1	0	5SNPs	0.6

aa, amino acid; S, susceptible; R, resistant/resistance. * S297T/F495I/G138C/M220T.

**Table 5:** *A. fumigatus* isogenic clinical isolates obtained from three independent patients with same MIC profile but different Hmg1 modifications.

Strain	Year	Origin	MICs (mg/L)				TRESPERG Genotype	Gene Modifications		
			ITC	VCZ	POS	ISV		Cyp51A	Hmg1	Hmg2
CM9114	2017	Barcelona	16	0.5	2	0.5	t05m1.8c22.e20	G54R	Y564H	WT
CM9501	2018	Barcelona	16	0.5	2	0.5	t05m1.8c22.e20	G54R	WT	WT
CM9551	2019	Barcelona	16	0.5	2	0.5	t05m1.8c22.e20	G54R	g799a	WT

ITC, itraconazole; VCZ, voriconazole; POS, posaconazole; ISV, isavuconazole.

**Table 6:** Specific mutations in the SSD domain of Hmg1 that have been introduced in a wild type azole-susceptible *A. fumigatus* strain and the MICs to azoles and AmB of the *A. fumigatus* mutants created.

HMG1 MUTATION	MICs (mg/L)					Reference
	ITC	VRC	POS	ISV	AmB	
**F262del**	32	8	2	8	-	[23]
*akuB*^KU80^, F262del	1	1	0.25	4	-	
**S269F**	>8	>8	8	-	1	[17]
*hmg1* ^S269F^	0.5–1	0.5	2–4	-	1–2	
*hmg1* ^WT^	1	1	1–4	-	2	
**S269F**	>8	>8	4	-	-	[25]
Δ*hmg1*^S269F^::*hmg1*^wild^	1	1	0.5–1	-	-	
Δ*hmg1*^S269F^::*hmg1*^S269F^	>8	>8	4	-	-	
**S305P**	>32	16	1	-	-	[23]
*akuB*^KU80^ S305P	0.5	2	0.25	4	-	
**S305P**	>8	>8	8	-	-	[25]
*cyp51A*^M220I^,Δ*hmg1*^S305P^::*hmg1*^wild^	>8	1–2	4	-	-	
*cyp51A*^M220I^,Δ*hmg1*^S305P^::*hmg1*^S305P^	>8	>8	8	-	-	
**E306K**	>16	4	2	-	0.125	[30]
*akuB*^KU80^ E306K	>16	4	2	-	0.125	
**G307D**	>8	>8	>8	-	-	[25]
*cyp51A*^M220I^,Δ*hmg1*^G307D^::*hmg1*^wild^	>8	>8	>8	-	-	
*cyp51A*^M220I^,Δ*hmg1*^G307D^::*hmg1*^G307D^	>8	>8	>8	-	-	
**I412S**	>32	8–16	0.5–1	-	-	[23]
*akuB*^KU80^ I412S	1	1	0.5	4	-	

## Data Availability

All sequence data and protocols associated with the publication are available to readers on request.

## Supplementary Materials

The following are available online. Figure S1: Secondary structure and amino acid sequence of the predicted Hmg proteins. (a) Hmg1. (b) Hmg2. The sterol sensing domain (SSD) is marked in purple. Seven transmembrane domains are identified in both proteins. Table S1: Analysis of the Hmg1 amino acid substitutions found in our set of 170 *A. fumigatus* clinical strains. Table S2: Analysis of the Hmg2 amino acid substitutions found in our set of 170 *A. fumigatus* clinical strains. In bold the amino acids comprised in the SSD. Concomitant Cyp51A azole resistance mechanisms are also shown. 3SNPs and 5SNPs only confer an intermediate level of susceptibility to azoles. Table S3: Primers used for amplifying and sequencing the genes *cyp51A,* promoter, *cyp51B, hmg1* and *hmg2*. Table S4: Primers used in RT-qPCR assays for amplifying *cyp51A, cyp51B, hmg1, hmg2* and *tub1* genes.

## Sample Availability

Strains are available to readers on request.
