# Supplementary material for: Are Point Mutations in HMG-CoA Reductases (Hmg1 and Hmg2) a Step towards Azole Resistance in Aspergillus fumigatus?
Source: Molecules. 2021 Oct 1;26(19):5975. doi: 10.3390/molecules26195975 (PMC8512156; doi:10.3390/molecules26195975)
Supplement: Supplementary file 1 [file molecules-26-05975-s001.zip › molecules-1394861-supplementary.pdf]

Supplementary material

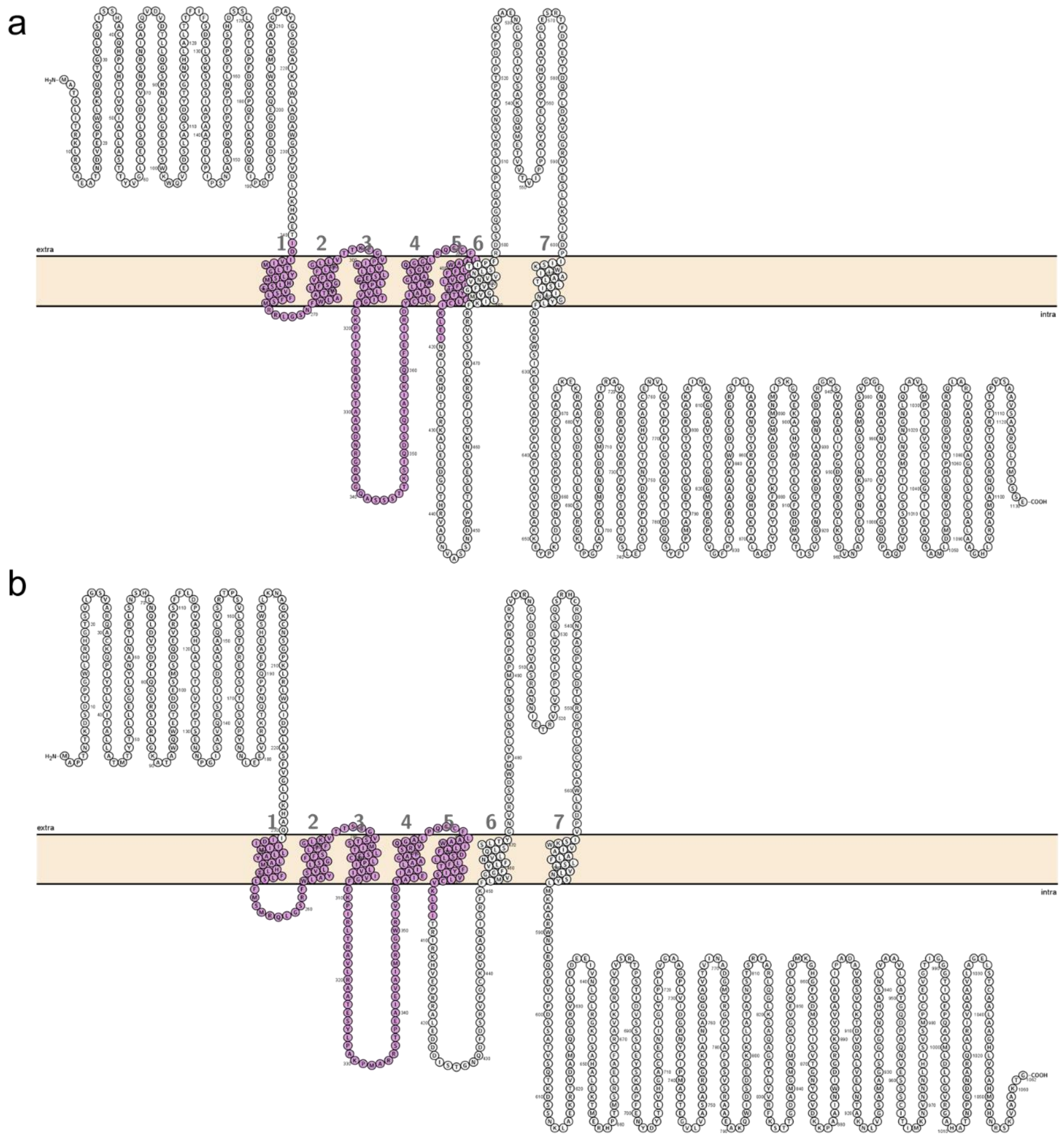

Figure S1: Secondary structure and amino acid sequence of the predicted Hmg proteins. (a) Hmg1. (b) Hmg2. The sterol sensing domain (SSD) is marked in purple. Seven transmembrane domains are identified in both proteins.

Table S1: Analysis of the Hmg1 amino acid substitutions found in our set of 170 *A. fumigatus* clinical strains.

Nucleotide position (cDNA)	Codon change	AA change	Nº of strains	Azole S	Azole R	Cyp51A modifications	Percentage (%)
313	Gag/Aag	E105K	10	1	9	TR34/L98H, G138C	5.8
634	Tcg/Ccg	S212P	1	1	0	--	0.5
919	Ggc/Agc	G307S	4	0	4	G138C	2.4
955	Aag/Cag	K319Q	1	0	1	M220I/V101F	0.5
1102	Tac/Cac	Y368H	1	1	0	P216L	0.5
1235	aTc/aCc	I412T	2	2	0	--	1.17
1454	tTt/tCt	F485S	1	0	1	P216L	0.5
1621	Agc/Ggc	S541G	8	4	4	M220T, G54E,TR34/L98H	4.7
1690	Tac/Cac	Y564H	15	15	0	--	8.8

S: susceptible; R: resistant/resistance; AA: amino acid. Substitutions marked in grey are located inside the SSD.

Table S2: Analysis of the Hmg2 amino acid substitutions found in our set of 170 *A. fumigatus* clinical strains. In bold the amino acids comprised in the SSD. Concomitant Cyp51A azole resistance mechanisms are also showed. 3SNPs and 5SNPs only confer an intermediate level of susceptibility to azoles.

Nucleotide (cDNA)	Codon change	aa change	N° of strains	Azole S	Azole R	Cyp51A modifications	Percentage (%)
32	aCt/aTt	T11I	1	1	0	-	0.6
466	Aca/Gca	T156A	5	4	1	3SNPs	2.9
514	Tct/Cct	S172P	6	6	0	3SNPs	3.5
704	aTc/aGc	I235S	3	2	1	M220V	1.8
908	gTa/gCa	V303A	15	5	10	TR_{34}/L98H/+*	8.8
935	aTc/aGc	I312S	4	3	1	3SNPs	2.4
1078	Att/Ttt	I360F	10	0	10	TR_{34}/L98H/+*	5.9
1190	tAc/tGc	Y397C	1	1	0	5SNPs	0.6
1298	gAc/gGc	D433G	2	0	2	TR_{34}/L98H,G54E	1.2
1343	cGg/cAg	R448Q	1	1	0	5SNPs	0.6
1499	gTa/gCa	V500A	34	27	7	3SNPs, G54R/E	20
1620	aaC/aaG	N540K	1	0	1	TR_{34}/L98H	0.6
1948	Cat/Tat	H650Y	3	3	0	-	1.8
2027	cGc/cAc	R676H	2	2	0	-	1.2
2048	cCg/cAg	P683Q	3	0	3	TR_{34}/L98H/+*	1.8
2332	Tgt/Cgt	C778R	1	1	0	-	0.6
2521	Ggg/Tgg	G841W	1	1	0	5SNPs	0.6
2723	aAg/aTg	K908M	3	0	3	TR_{34}/L98H/+*	1.8
3092	gCa/gTa	A1031V	1	1	0	5SNPs	0.6

aa, amino acid; S, susceptible; R, resistant/resistance. * S297T/F495I/G138C/M220T. Substitutions marked in grey are located inside the SSD.

Table S3: Primers used for amplifying and sequencing the genes *cyp51A,* its promoter, *cyp51B, hmg1* and *hmg2*.

Gene	Primer name	Sequence (5′-3′)	Tm	Application	Origin
cyp51A Promoter	A5R	TCTCTGCACGCAAAGAAGAAC	56°C	PCR and sequencing	[14]
	A7F	TCATATGTTGCTCAGCGG			
cyp51A	P450.1F	ATGGTGCCGATGCTATGG	58°C	PCR	
	P450.2R	CTGTCTCACTTGGATGTG			
	A1F	CTTCTTTGCGTGCAGAGA		Sequencing	
	A3F	TAGTCCATTGACGACCCC		Sequencing	
	A4F	CAGACATGATATGGAACC		Sequencing	
	A10R	ATTGCCGCAGAGATGTCC		Sequencing	
	Afer12R	GGGAGGAATCATGTAAGGGG		Sequencing	
	CypA1F	CTTACGGCCTACATGGCC		Sequencing	
	CypA2R	TTCGACCGCTTCTCCCAG		Sequencing	
cyp51B	P450.3F	ATGGGTCTCATCGCGTTC	58°C	PCR and sequencing	
	P450.4R	TCAGGCTTTGGTAGCGG			
	B1.F	CTTTTTCGACTGCCGCGC		Sequencing	
	B2.R	AGGCGTAGTGAGTGGAGA		Sequencing	
	B3.F	TCTCCACTCACTACGCCT		Sequencing	
	B5.R	TTGCGCGGCAGTCGAAAAAGAAC		Sequencing	
	B6.F	CATGGCTGTGGATGGTACTTC		Sequencing	
	B8.F	CAGTGAAGAATCCCATGG		Sequencing	
	Afer7.R	CCARCGRTGNGGRTCCCA		Sequencing	
hmg1	P1	TGCTATCAAGCTCTGGTTGG	60°C	PCR and sequencing	This study
	P2	CAGAGGCCAATCATTACTGG			
	P3	GCTACCTCTCTGATTACAAGG	55°C		
	P4	GCCAACCAGAGCTTGATAGC			
	P5	GAAGATGGTATCACCCATCGG	60°C		
	P6	CTCGCTTCTCTTTCAAGAAGG			
	P7	CACTCCCAAGAGGAGTTTAG	56°C		
	P8	CACTCAGTCGCCATAACGTGG			
	P9	GGCACTTATCTTTACATCCGG	60°C		
	P10	CGAGGAGGACATCGTCAATC			
hmg2	P1	ATGCAATTCTGGTCCGAAGC	63°C	PCR and sequencing	
	P2	TTGACCTTGCAGCCAAAGAC			
	P3	AAGACTCTGACACTCCTGG	55°C		
	P4	GCTGGCGCATTGACATAAAG			
	P5	GACGATGACATAAGCACTGG	59°C		
	P6	CTCTGGTAAAGGCTTCCAAGC			
	P7	GGAGATAGTCAATCTATGCC	53°C		
	P8	GTTCTTGTCGACGCAGTAG			
	P9	CTTGAAGTCATGAAGGGGC	61°C		
	P10	TTGCGGTTCAAGGATGGTTC			

Tm: melting temperature

Table S4: Primers used in RT-qPCR assays for amplifying *cyp51A, cyp51B, hmg1, hmg2* and *tub1* genes.

Gene	Primer name	Sequence (5'-3')
cyp51A	A1	CTTCTTTGCGTGCAGAGA
	A10	ATTGCCGCAGAGATGTCC
cyp51B	B1	CTTTTTCGACTGCCGCGC
	B2	AGGCGTAGTGAGTGGAGA
hmg1	RT5	TTCAACGCTCACGCATCGAC
	RT6	ACCTAGCATATCAAGCATGGC
hmg2	RT7	TCCATGCATCCAATCTGGTGG
	RT8	TTGCGGTTCAAGGATGGTTC
tub1	Tub5	TGACCCAGCAGATGTT
	Tub6	GTTGTTGGGAATCCACTC
